# Layers of Uranium Phosphate Nanorods and Nanoplates Encrusted on Fungus *Cladosporium* sp. Strain F1 Hyphae

**DOI:** 10.1264/jsme2.ME21036

**Published:** 2021-11-13

**Authors:** Jisu Lee, Sue Jung Lee, Sungho Kim, Jong-Un Lee, Kwang-Soon Shin, Hor-Gil Hur

**Affiliations:** 1 School of Earth Sciences and Environmental Engineering, Gwangju Institute of Science and Technology, Gwangju 61005, Republic of Korea; 2 Department of Food Science and Biotechnology, Kyonggi University, 154–42, Gwanggyosan-ro, Youngtong-gu, Suwon, Gyeonggi 16227, Republic of Korea; 3 GIST Central Research Facilities, Gwanju Institute of Science and Technology, Gwangju 61005, Republic of Korea; 4 Department of Energy and Resources Engineering, Chonnam National University, Gwangju 61186, Republic of Korea

**Keywords:** uranium, nanoplate, fungus, biosorption, exopolysaccharides

## Abstract

The hyphae of *Cladosporium* sp. strain F1 (CFGR 2020-301-00084) were heavily encrusted with pre-synthesized uranium phosphate minerals under a wide range of pH conditions. SEM and TEM images showed that nanorods and nanoplates of uranium phosphate minerals at pH 4 and 5 and at pH 6, 7, and 8, respectively, were tightly adsorbed along the hyphae of *Cladosporium* sp. strain F1, while only a few uranium phosphate minerals were observed on the hyphae of *Aspergillus niger* VKMF 1119. Based on the physical mobility and chemical stability of uranium phosphate minerals under *in situ* oxidizing environmental conditions, the application of *Cladosporium* sp. strain F1 has potential as a novel strategy for the remediation of uranium contamination in sediments and aquifers under a wide range of pH conditions where larger amounts of phosphate are present in the environment.

The long-lived radioactive and chemically toxic element uranium acts as a mutagenic, carcinogenic, neurotoxic, and nephrotoxic chemical that affects the human kidneys, liver, lungs, and hematopoietic system, ultimately leading to death (Arzuaga *et al.*, 2010; Hon *et al.*, 2015). Although the ionizing radiation toxicity of uranium generally draws public concerns, uranium risks associated with chemical toxicity may be greater than those of radiotoxicity depending on its compound, composition, or enrichment grade (Rump *et al.*, 2019). The chemical toxicity of uranium also depends on its oxidation state. Uranium predominantly exists in the environment in two oxidation states, tetravalent and hexavalent. Tetravalent uranium (U[IV]) is less soluble and mobile in the aqueous phase than hexavalent uranium (U[VI]) (Anderson, 1984). The toxicity of more soluble U(VI) is generally greater than that of less soluble U(IV) (Wufuer *et al.*, 2017). By using differences in the solubility and mobility of the two oxidation states of uranium, various methods, including anaerobic bacterial respiration systems, have been proposed to remove U(VI) from the environment (Jiang *et al.*, 2011; Li and Zhang, 2012; Jiang and Hur, 2013; Lee and Hur, 2014; Parth *et al.*, 2014). However, it is important to note that reduced U(IV) may be easily re-oxidized to soluble U(VI) under oxidized field conditions (Casas *et al.*, 1994; Abdelouas *et al.*, 1999; Gu *et al.*, 2005; Zhong *et al.*, 2005; Zhou and Gu, 2005; Moon *et al.*, 2009).

Therefore, a novel method to immobilize U(VI) species that considers physical mobility and chemical stability under oxidized conditions is needed. Uranium phosphate minerals may meet these conditions even in fluid-rich oxidizing environments. Phosphates play an important role in the immobilization of trace elements, including uranium, through secondary phosphate mineral precipitation in well-developed and fluid-rich weathering profiles (Banfield and Eggleton, 1989; Braun *et al.*, 1998; Taunton *et al.*, 2000; Mehta *et al.*, 2013; Kanematsu *et al.*, 2014). A number of uranium phosphate minerals have been observed in environments in which U(VI) and phosphate exist. Copper meta-torbernite (Cu[UO_2_]_2_[PO_4_]_2_·8H_2_O) and magnesium meta-autunite (Mg[UO_2_]_2_[PO_4_]_2_·*n*H_2_O) were detected near the Koongarra uranium deposit in Australia, and barium meta-autunite (Ba[UO_2_]_2_[PO_4_]_2_·*n*H_2_O) in the vicinity of the Coles Hill uranium deposit in Virginia, USA (Murakami *et al.*, 1997; Jerden *et al.*, 2003; Murakami *et al.*, 2005; Jerden and Sinha, 2006). Due to the low solubility and high stability of uranium phosphate minerals under oxidizing conditions, the addition of phosphate to environments containing uranium has been suggested for its removal (Fanizza *et al.*, 2013; Munasinghe *et al.*, 2015; Baker *et al.*, 2019). However, precipitated uranium phosphate minerals may cause other threats. Liu *et al.* (2015) reported that uranium phosphate particles entered normal rat hepatic BRL cells and induced apoptotic cell death. Therefore, methods with the ability to collect and remove stable uranium phosphate minerals from the environment are desired.

Various organisms, including bacteria, fungi, algae, and plants, may be used as biomasses to adsorb heavy metals. Biosorption using a fungal biomass is considered to be more effective than that by other biomasses for a number of reasons including the high binding capacity associated with the large surface area of fungal hyphae, the ease of cultivating fungi on a large scale, and the use of inexpensive growth media (Dhankhar and Hooda, 2011). Therefore, many fungal biomasses, such as *Aspergillus*, *Fusarium*, *Mucor*, *Penicillium*, *Rhizopus*, *Talaromyces*, and *Trichoderma*, have been examined and identified as promising biosorbents to remove uranium in the environment (Gupta *et al.*, 2018). Although *Cladosporium* is one of the most common fungi worldwide, the biosorption of uranium phosphate minerals by this fungal biomass has not yet been reported.

In the present study, we report the unusual adsorption phenomenon of the newly isolated fungus *Cladosporium* sp. strain F1 towards uranium phosphate minerals. The hyphal surface of strain F1 formed tight and extensive layers of nanorods and nanoplates made of uranium phosphate minerals, which may be used to collect and remove uranium in the form of stable uranium phosphate minerals from the environment. The role of fungal exopolysaccharides (EPSs) in uranium phosphate mineral binding was examined and characterized.

## Materials and Methods

### Fungal strains, uranium preparation, and experiments conditions

*Cladosporium* sp. strain F1 was isolated from contaminated solution containing U(VI) and phosphate in our laboratory, and deposited in the Center for Fungal Genetic Resources (Seoul, Korea) with the strain number CFGR 2020-301-00084. It was identified by its internal transcribed spacer (ITS) sequence, registered with the National Center for Biotechnology Information (Bethesda, MD, USA), and obtained the accession number of the ITS sequence of MT271897. *Aspergillus niger* VKMF 1119 was kindly provided by Dr. C. Cerniglia, the National Center for Toxicological Research (Jefferson, AR, USA). Both fungi were grown in potato dextrose broth (PDB) at 25°C with 150‍ ‍rpm for 7‍ ‍d prior to experimental subculture.

To prepare uranium phosphate minerals, 1.97‍ ‍mg of uranyl nitrate was added to 50‍ ‍mL of aqueous solution containing potassium dihydrogen phosphate (0.14‍ ‍mM) and disodium hydrogen phosphate (0.21‍ ‍mM). The solution pH was varied from pH 4 to 8. After uranium phosphate minerals had been synthesized and precipitated in solution at room temperature for 24 h, the fungal biomass was added. The fungus weight was 50‍ ‍mg of dry weight per 50‍ ‍mL of solution. All biosorption experiments were performed at 25°C with shaking at 150‍ ‍rpm and conducted in duplicate.

After adsorbing uranium phosphate minerals to the hyphae of the two fungal biomasses for 12 h, samples were filtered using a 5-μm nylon net filter (Merck Millipore) to separate uranium phosphate minerals adsorbed on fungal hyphae and those remaining in solution. Filtrates, which contained the remaining uranium phosphate minerals, were centrifuged at 10,000×*g* for 10‍ ‍min. After centrifuging, uranium phosphate pellets were solubilized by acid treatments using 5‍ ‍mL of concentrated nitric acid. Total uranium concentrations were assessed by inductively coupled plasma mass spectrometry (ICP-MS, 1100 & 7500ce; Agilent Technologies). The kinetics of fungal biosorption toward uranium phosphate minerals were also examined by ICP-MS. Filtrates were collected after reacting for 1, 3, and 6 h at pH 7 following the above process. Biomineralization experiments were performed with the two fungal strains in 10‍ ‍mL MOPS buffer (10‍ ‍mM) at 25°C with shaking at 150‍ ‍rpm. MOPS buffer containing sucrose at 50‍ ‍mM, glycerophosphate at 2‍ ‍mM, and uranyl nitrate at 0.4‍ ‍mM was adjusted to pH 6. Experiments were conducted in duplicate.

### Identification of the isolated fungal strain

The isolated fungal strain was identified using ITS sequence analyses. The ITS region was amplified by a polymerase chain reaction (PCR) using the following primers: ITS1 (5′-TCCGTAGGTGAACCTGCGG-3′) and ITS4 (5′-TCCTCCGCTTATTGATATGC-3′). PCR was performed using the following conditions: initial denaturation step at 95°C for 5‍ ‍min, followed by 30 cycles at 95°C for 30‍ ‍s, 55°C for 120‍ ‍s, and 68°C for 90‍ ‍s, and a final extension step at 68°C for 10‍ ‍min. PCR products were purified using the Montage PCR Clean up kit (Merck Millipore). Purified PCR products were sequenced using Big Dye terminator cycle sequencing kit v.3.1 (Applied BioSystems) with the two primers above. Sequencing products were resolved on an Applied Biosystems model 3730XL automated DNA sequencing system (Applied BioSystems) at Macrogen. The elucidated sequences were aligned and a phylogenetic tree was constructed with the Clustal W program using the maximum likelihood method. The genetic distance of similar sequences was calculated using the MEGA 7 software program (Kumar *et al.*, 2016).

### Isolation of EPSs

*Cladosporium* sp. strain F1 and *A. niger* VKMF-1119 were grown in PDB at 25°C for 7 d. Cultures were centrifuged at 10,000×*g* for 10‍ ‍min and the supernatant was filtered using a 10-μm nylon net filter (Merck Millipore). A four-fold volume of 95% (v/v) ethanol was added and the mixture was kept at 4°C overnight. The resulting precipitates were recovered by centrifugation at 10,000×*g* for 10‍ ‍min and desalted using Amicon Ultra-15 10K centrifugal filter devices (Merck Millipore). Samples were lyophilized and used in subsequent experiments.

### Structural characterization of EPSs

In polysaccharide samples, total carbohydrate, uronic acid, 2-keto-3-deoxy-D-*manno*-octulosonic acid (KDO)-like materials, and protein contents were measured using the phenol-sulfuric acid method, *m*-hydroxybiphenyl method, modified thiobarbituric acid method, and Bradford method (Bio-Rad Laboratories), respectively (Blumenkrantz and Asboe-Hansen, 1973; Bradford, 1976; Karkhanis *et al.*, 1978). The molecular weight (MW) profiles of polysaccharide samples were analyzed by high performance size-exclusion chromatography (HPSEC) using an Agilent 1260 Infinity LC system (Agilent Technologies) according to the method described by Lee *et al.* (2014). Sugar compositions elucidated using the 1-phenyl-3-methyl-5-pyrazolone (PMP) derivative method were analyzed at 30°C by high performance liquid chromatography (HPLC) (Shimadzu) equipped with a SPD-20A UV/VIS detector and Acclaim^TM^120 C18 column (Thermo Fisher Scientific) (Honda *et al.*, 1989; Dai *et al.*, 2010). The elution of PMP derivatives was performed using a mixture of phosphate buffer (82%, 0.1 M, pH 6.7) and acetonitrile (18%) at a flow rate of 1‍ ‍mL‍ ‍min^–1^. The UV absorbance of the eluate was monitored at 245‍ ‍nm.

A methylation analysis of glycosidic linkages within polysaccharides was performed using previously reported methods with minor modifications (Hakomori, 1964; Pettolino *et al.*, 2012). In brief, dimethyl sulfoxide (DMSO) and methylsulfinyl carbanion with iodomethane were used to dissolve the polysaccharide fraction for the methylation of polysaccharides. Unmethylated parts were removed using a Sep-pak C18 cartridge (Waters), and the methylated sample was then hydrolyzed with 1 M trifluoroacetic acid at 121°C for 90‍ ‍min. Sodium borodeuteride was used to reduce hydrolyzed-methylated samples for 240‍ ‍min. Samples were then acetylated with acetic anhydride at 121°C for 180‍ ‍min. Gas chromatography-mass spectrometry (GC-MS, Agilent Technologies) was used to analyze the resulting partially methylated alditol acetates (PMAAs). Glycosidic linkages in the polysaccharide fraction were counted based on the method described by Lee *et al.* (2018).

### Morphological and mineralogical analyses

Pre-synthesized uranium phosphate minerals were collected after 24 h for morphological and mineralogical analyses. The fungal biomass and EPSs were collected after reacting with pre-synthesized uranium phosphate minerals for 12 h. The samples collected were centrifuged at 10,000×*g* for 10‍ ‍min, and then washed three times with sterile distilled water using centrifugation.

Washed samples were analyzed using scanning electron microscopy (SEM, SU-70; Hitachi), transmission electron microscopy (TEM, Tecnai G2 F30 S-Twin; Field Electron and Ion Company), atomic force microscopy (AFM, XE-100; Park Systems), and X-ray diffraction (XRD, D8 advance; Bruker). In SEM and AFM analyses, 5–10‍ ‍μL of samples were placed on silicon wafers and air-dried. In TEM analyses, samples were mounted on copper grids and subsequently air-dried. Energy dispersive X-ray spectroscopy (EDX) was used with SEM and TEM analyses, and selected area electron diffraction (SAED) was equipped at TEM. XRD analyses were performed with fine powder samples. In cross-sectional TEM analyses, washed cells were fixed with 3% (v/v) glutaraldehyde for 2 h and 1% osmium tetroxide (OsO_4_) at 4°C for 3 h and then dropped onto mesh copper grids covered with carbon film. Cross-sectioned specimens were prepared using a microtome (CR-X; RMC Products). Images were obtained at 200 kV using JEOL JEM-2100 high resolution TEM (JEOL). In morphological analyses of uranium phosphate precipitates after biomineralization experiments, fungal biomasses were collected after 1, 2, 3, and 4‍ ‍weeks, and the samples collected were prepared as described above.

### Analytical methods

ICP-MS (1100 & 7500ce; Agilent Technologies) was used to quantify uranium. One milliliter of samples was filtered through a 0.22-μm syringe filter (Whatman), and diluted using high-purity 2% (v/v) HNO_3_.

## Results and Discussion

### Identification of the isolated fungus

In fungal biomineralization experiments performed using the newly isolated fungus strain F1, SEM images showed the precipitation of minerals on the hyphae of the fungus, which was different from *A. niger* VKMF-1119 (Fig. S1). EDX revealed that the minerals that precipitated on fungal hyphae were made of uranium and phosphate. Based on these results, we assumed that an enzyme phosphatase catalyzed the release of phosphate from glycerophosphate added to the solution, which then reacted with uranium to form uranium phosphate minerals. The activity of acid phosphatase, which was excreted into the fungal agar medium, was confirmed by a color reaction (Matos *et al.*, 2017). In addition, the isolated fungal strain was phylogenetically identified as the genus *Cladosporium* (Fig. S2), named *Cladosporium* sp. strain F1 with the accession number MT271897 of the ITS sequence.

### Characterization of pre-synthesized uranium phosphate minerals

**Fig. 1.**
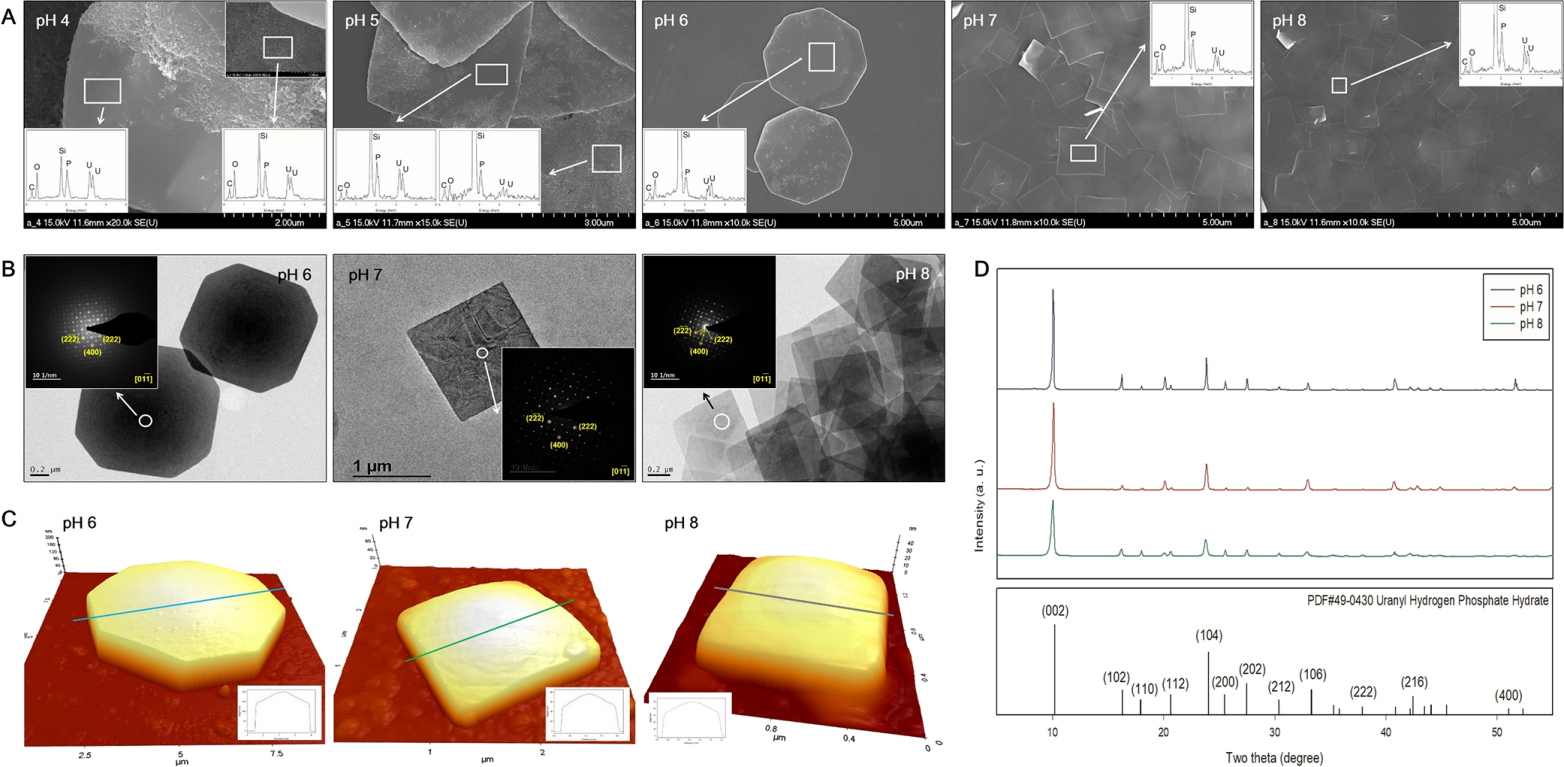
SEM images of uranium phosphate minerals synthesized from pH 4 to 8 with EDX results (A, inset), TEM images with SAED patterns (B, inset), AFM images with thickness profiles (C, inset), and XRD results (D) of uranium phosphate nanoplates synthesized from pH 6 to 8.

SEM images showed that the nanoplate shapes of uranium phosphate minerals were mostly detected at pH 4 to 8 (Fig. 1A). In addition, as pH decreased from 7 to 4, nanorod-shaped minerals increased. SEM images also showed that as pH increased from 6 to 8, octagonal-shaped uranium phosphate nanoplates gradually changed to tetragonal-shaped nanoplates. The length of each uranium phosphate nanoplate synthesized at pH 6, 7, and 8 ranged from 3–4, 1–2, and 0.5–1‍ ‍μm, respectively. TEM-SAED pattern analyses showed distinct electron diffraction patterns with the high crystallinity of nanoplates for uranium phosphate nanoplates synthesized at pH 6, 7, and 8 (Fig. 1B). The two diffractions angled 45° had interplanar distance values of 2.374 and 1.746 Å respectively, which were indexed as the crystal planes of (222) and (400) of the p4/ncc structure (inset, Fig. 1B). The crystallinity of the materials was confirmed by XRD patterns in the high angle region (Fig. 1D). A series of peaks were detected at 2θ=10.192°, 16.287°, 17.938°, 20.634°, 24.032°, 25.480°, 27.481°, 30.378°, 33.305°, 37.867°, 42.423°, and 52.358° for the samples prepared at pH 6, 7, and 8, which corresponded to reflections by (002), (102), (110), (112), (104), (200), (202), (212), (106), (222), (216), and (400) planes, respectively. This result is consistent with SAED patterns. Therefore, we assumed that the precipitated uranium phosphate mineral was uranyl hydrogen phosphate hydrate (HPUO_6_·4H_2_O), which matched the reference patterns with PDF#49-0430 from Powder Diffraction Files.

AFM analyses showed that the central point of each nanoplate was the thickest and thickness gradually decreased towards the edge of the mineral under all pH conditions (inset) (Fig. 1C). As pH increased from 6 to 8, the height of each nanoplate at the center point decreased from approximately 203 to 50‍ ‍nm. Although it was not possible to explain the dependence of the precipitation of different shapes of uranium phosphate minerals on different pH conditions in the present study, it may be attributed to a change in the predominant aqueous uranium species under different pH conditions (Suzuki and Banfield, 1999).

### Adsorption of pre-synthesized uranium phosphate minerals to *Cladosporium* sp. strain F1 and *A. niger* VKMF-1119

**Fig. 2.**
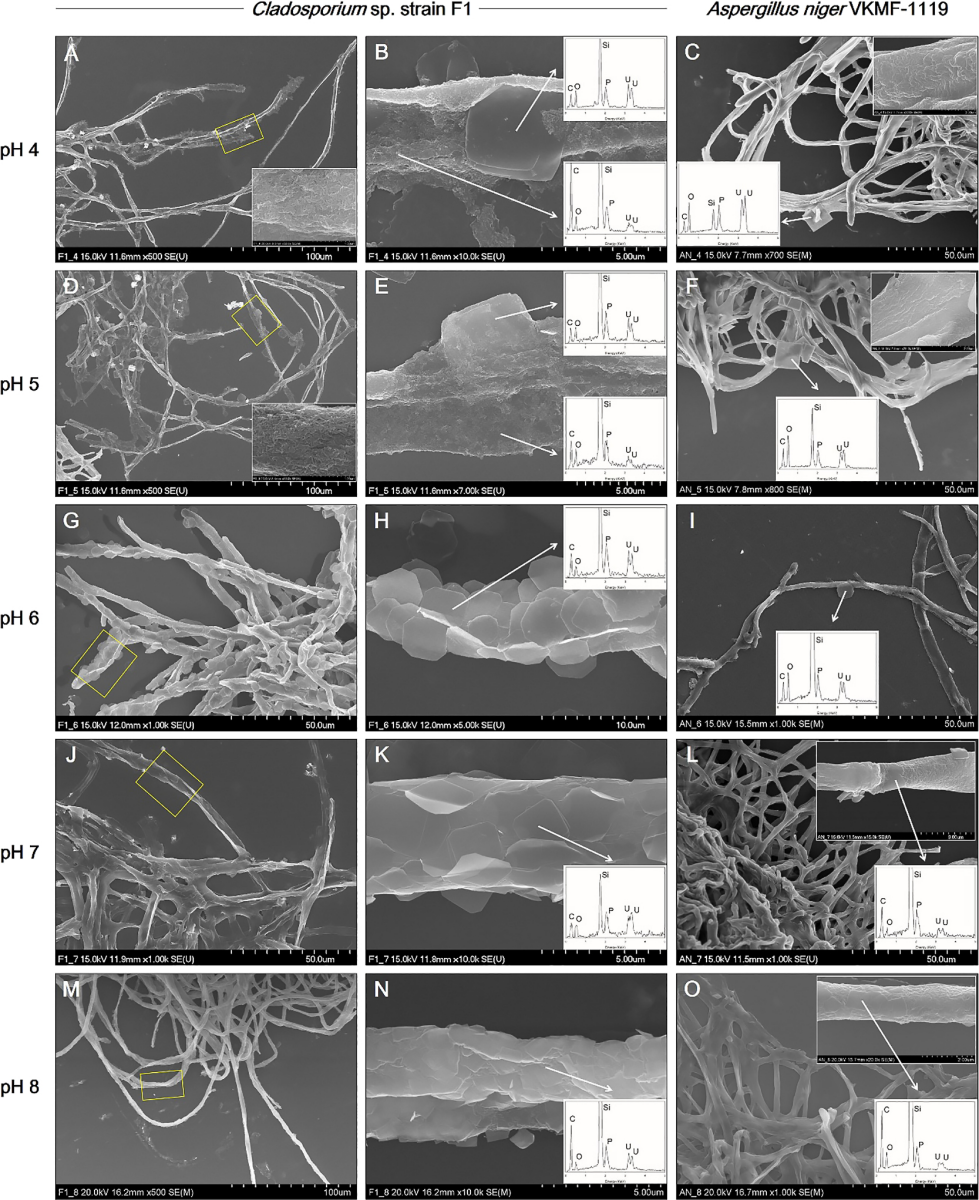
SEM images of uranium phosphate nanoplates adsorbed on hyphae of *Cladosporium* sp. strain F1 at pH 4 (A and B), 5 (D and E), 6 (G and H), 7 (J and K), and 8 (M and N), and on hyphae of *Aspergillus niger* VKMF-1119 at pH 4 (C), 5 (F), 6 (I), 7 (L), and 8 (O) with EDX results (inset). The adsorbed uranium phosphate nanorods were also shown at pH 4 (inset at A and C) and 5 (inset at D and F).

*Cladosporium* sp. strain F1 and *A. niger* VKMF-1119 showed different patterns for adsorbing uranium phosphate minerals on their hyphae under the applied pH conditions (Fig. 2). At pH 4 and 5, *Cladosporium* sp. strain F1 hyphae were extensively encrusted with the nanorods and nanoplates of uranium phosphate minerals, while the adsorption of uranium phosphate minerals on *A. niger* VKMF-1119 was limited to a few nanoplates (Fig. 2A, B, C, D, E, and F). In addition, *Cladosporium* sp. strain F1 hyphae were encrusted with multiple layers of uranium phosphate nanoplates at pH 6, 7, and 8, whereas *A. niger* VKMF-1119 hyphae only adsorbed a few uranium phosphate nanoplates (Fig. 2G, H, I, J, K, L, M, N, and O). While the uranium phosphate nanoplates synthesized at pH 6 were adsorbed on the hyphae of *Cladosporium* sp. strain F1 with a rigid structure, nanoplates at pH 7 and 8 were bent and adsorbed along fungal hyphae with multiple layers. This may be explained by the size and thickness of each uranium phosphate nanoplate under the applied pH conditions. Therefore, the small and thin nanoplates that formed at pH 7 and 8 may lead to the extensive adsorption of uranium phosphate minerals to fungal hyphae.

**Fig. 3.**
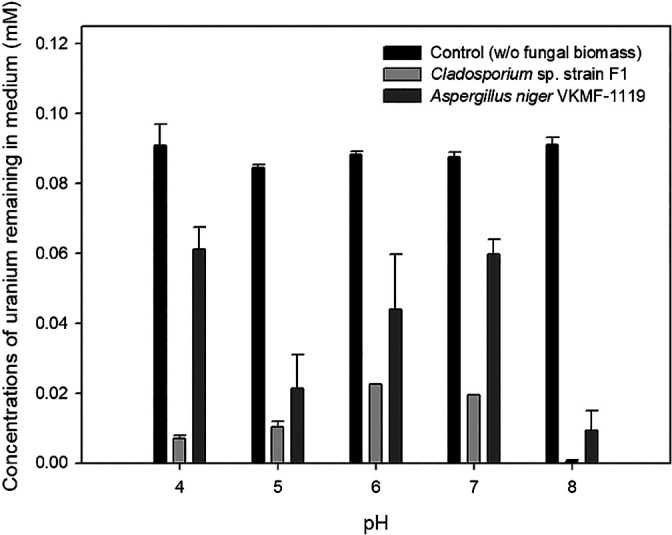
Amount of uranyl ions remaining in culture medium under different pH conditions.

To quantify the uranium phosphate minerals adsorbed on the hyphae of two fungal strains, solubilized uranyl ions from the uranium phosphate minerals remaining in medium were assessed by ICP-MS after filtering and acid treatments (Fig. 3). Although uranium concentrations decreased for *Cladosporium* sp. strain F1 and *A. niger* VKMF-1119, the amount of adsorbed uranium phosphate minerals markedly differed. *Cladosporium* sp. strain F1 adsorbed 93.1, 89.7, 77.5, 80.6, and 99.6% of uranium phosphate minerals at pH 4, 5, 6, 7, and 8, respectively, while only 38.8, 78.7, 56.1, 40.3, and 90.7% of minerals were adsorbed on *A. niger* VKMF-1119. These results quantitatively proved that *Cladosporium* sp. strain F1 adsorbed uranium phosphate minerals more efficiently than *A. niger* VKMF-1119, and, hence, represents a better option for the absorbance of uranium phosphate minerals under a wide range of pH conditions.

**Fig. 4.**
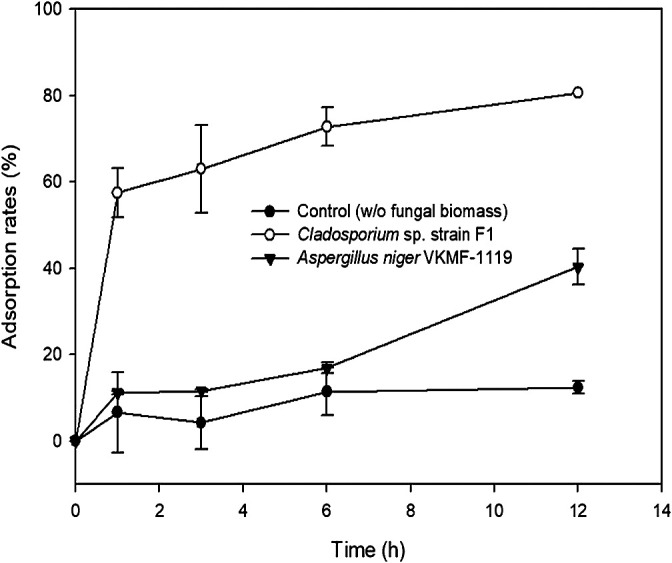
Adsorption kinetics of uranium phosphate minerals to hyphae of *Cladosporium* sp. strain F1 and *Aspergillus niger* VKMF-1119 with time at pH 7.

**Fig. 5.**
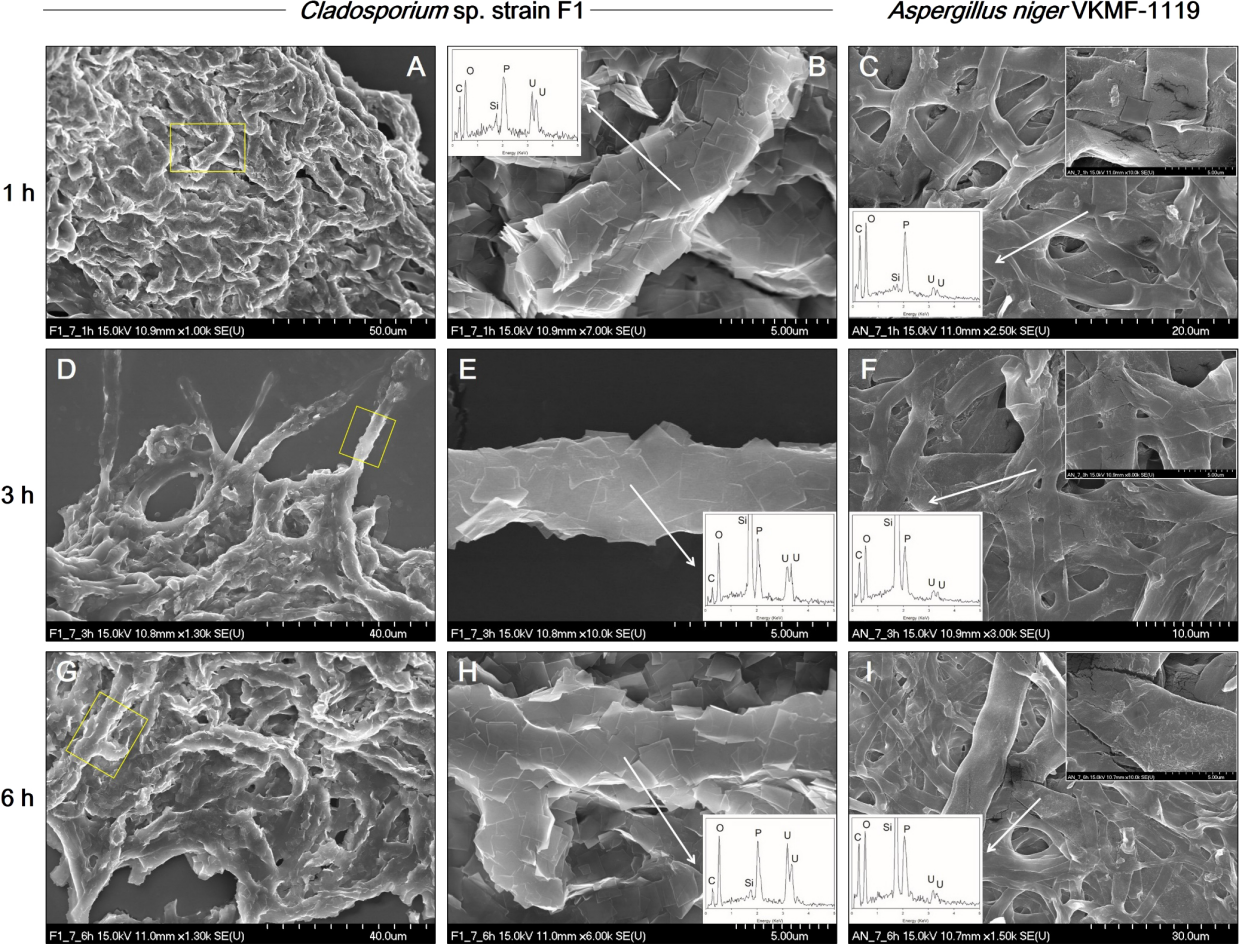
SEM images of uranium phosphate nanoplates adsorbed on hyphae of *Cladosporium* sp. strain F1 at 1 h (A and B), 3 h (D and E), and 6 h (G and H), and on hyphae of *Aspergillus niger* VKMF-1119 at 1 h (C), 3 h (F), and 6 h (I) at pH 7 with EDX results (inset).

The kinetics of fungal biosorption toward uranium phosphate minerals were quantitatively assessed by ICP-MS, and morphologically observed by SEM analyses at pH 7. ICP-MS results demonstrated that the biosorption capacity of *Cladosporium* sp. strain F1 toward uranium phosphate minerals was superior to that of *A. niger* VKMF-1119 (Fig. 4). *Cladosporium* sp. strain F1 adsorbed 57.5% of uranium phosphate minerals within 1 h, whereas 11.1% of minerals were adsorbed on *A. niger* VKMF-1119. Adsorbed uranium phosphate minerals increased to 63.0, 72.8, and 80.6% on the hyphae of *Cladosporium* sp. strain F1 after 3, 6, and 12 h, respectively, and only to 11.7, 16.9, and 40.3% on *A. niger* VKMF-1119. The amount of uranium phosphate minerals adsorbed on *Cladosporium* sp. strain F1 at 1 h (57.5%) was markedly higher than that on *A. niger* VKMF-1119 at 12 h (40.3%). Differences in biosorption capacities were also morphologically proven by SEM analyses (Fig. 5). *Cladosporium* sp. strain F1 hyphae were encrusted with uranium phosphate nanoplates at 1 h, whereas difficulties were associated with detecting nanoplates on the hyphae of *A. niger* VKMF-1119 (Fig. 5A, B, and C), and similar phenomena were observed at 3 h (Fig. 5D, E, and F) and 6 h (Fig. 5G, H, and I). Based on kinetic results, *Cladosporium* sp. strain F1 may be used as an effective adsorbent due to its fast and high biosorption capacity towards uranium phosphate minerals.

**Fig. 6.**
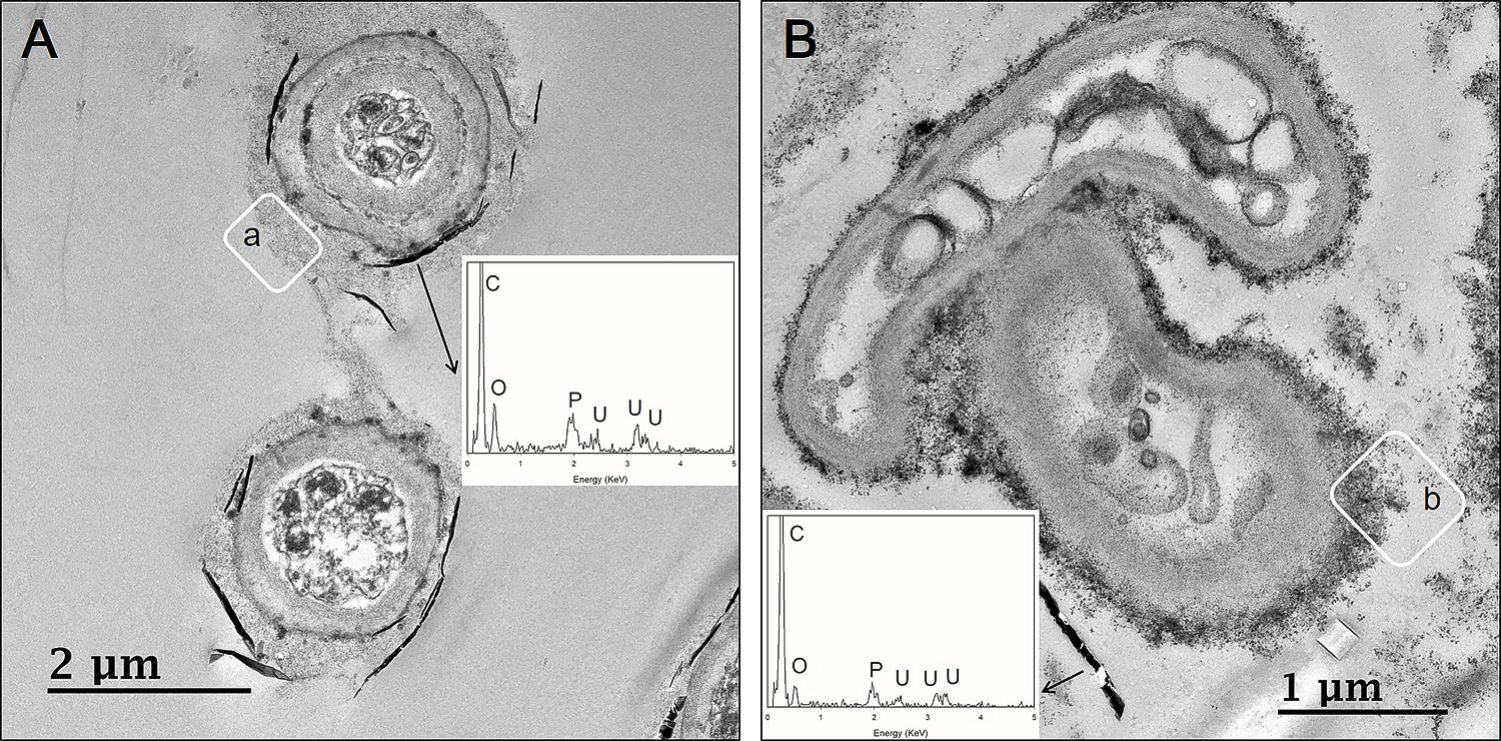
Cross-sectional TEM images of uranium phosphate nanoplates on hyphae of *Cladosporium* sp. strain F1 (A) and *Aspergillus niger* VKMF-1119 (B) at pH 7 with EDX results (inset).

To identify the cellular location of uranium phosphate minerals, cross-sectional TEM analyses were performed at pH 7. The results obtained showed that uranium phosphate minerals did not accumulate inside cells, they were only observed on the cell walls of fungal hyphae (Fig. 6). Therefore, pre-synthesized uranium phosphate minerals did not appear to have the ability to enter fungal cells. Cross-sectional TEM images also revealed that *Cladosporium* sp. strain F1 hyphae were encrusted by uranium phosphate nanoplates at pH 7 (Fig. 6A), while a few nanoplates were adsorbed on small portions of *A. niger* VKMF-1119 hyphae (Fig. 6B). Therefore, *Cladosporium* sp. strain F1 appeared to adsorb uranium phosphate minerals more effectively than *A. niger* VKMF-1119 at pH 7. TEM images revealed numerous grey-colored regions between the fungal cell wall and uranium phosphate nanoplates (inset, a and b) (Fig. 6). We assumed that these were fungal EPSs that link the fungal cell wall and uranium phosphate minerals.

### Adsorption of pre-synthesized uranium phosphate minerals to purified EPSs of *Cladosporium* sp. strain F1 and *A. niger* VKMF-1119

**Fig. 7.**
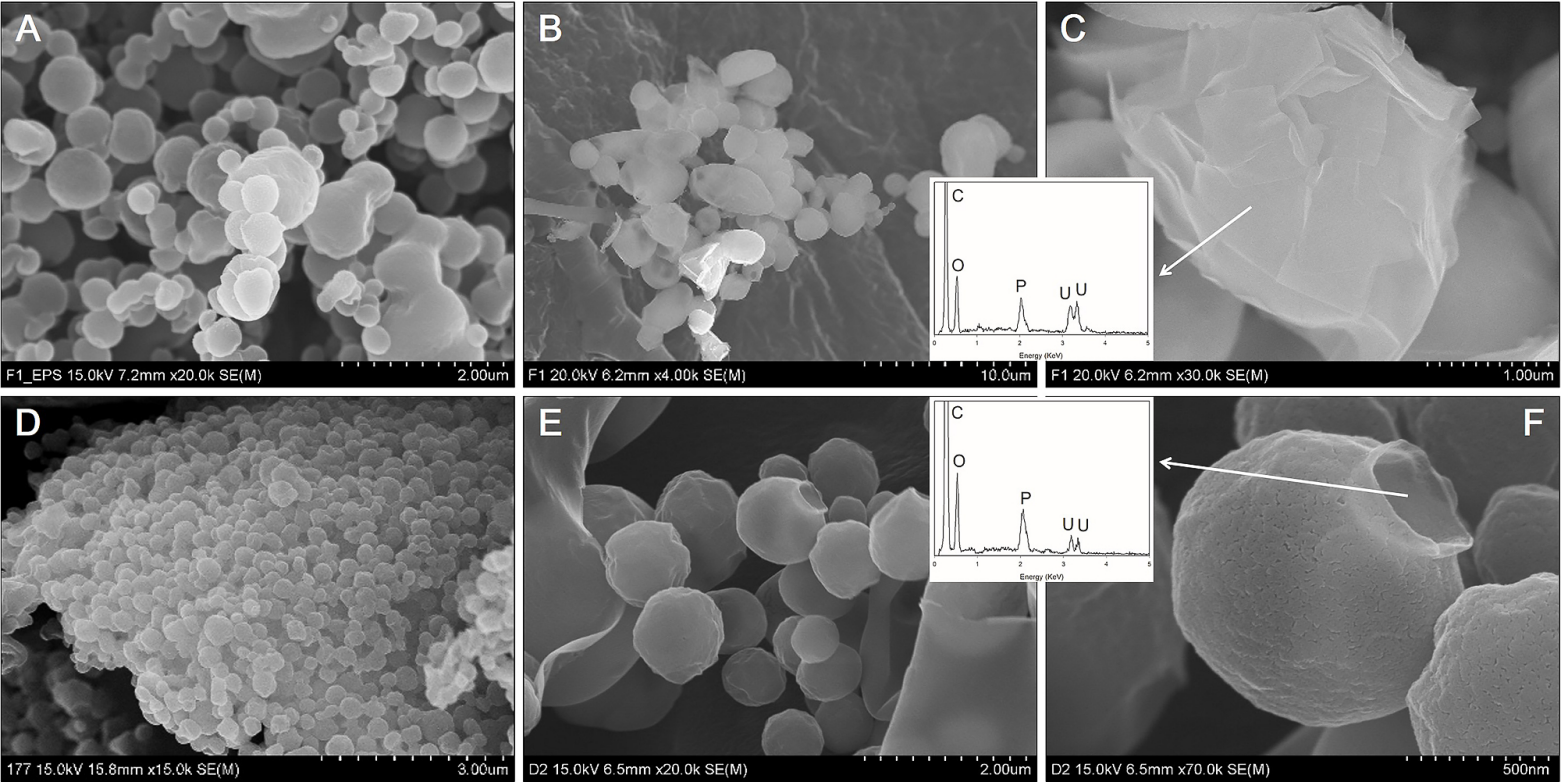
SEM images of EPSs from *Cladosporium* sp. strain F1 (A) and *Aspergillus niger* VKMF-1119 (D), and uranium phosphate nanoplates adsorbed on the surface of EPSs from *Cladosporium* sp. strain F1 (B and C) and *A. niger* VKMF-1119 (E and F) at pH 8 with EDX results (inset).

SEM images showed that purified EPSs from *Cladosporium* sp. strain F1 and *A. niger* VKMF-1119 after lyophilization were nanosized spheres with diameters of 0.2 to 2‍ ‍μm (Fig. 7A and D). Following the addition of 50‍ ‍mg of purified fungal EPSs to pre-synthesized uranium phosphate nanoplates in 50‍ ‍mL of solution at pH 8, the surfaces of EPSs from *Cladosporium* sp. strain F1 were compactly encrusted by uranium phosphate nanoplates (Fig. 7B and C), while EPSs from *A. niger* VKMF-1119 adsorbed few nanoplates (Fig. 7E and F). Therefore, fungal EPSs from different sources exhibited different hyphal binding capacities to uranium phosphate minerals possibly due to compositional differences in the structures of the two fungal EPSs.

### Characterization of EPSs produced by *Cladosporium* sp. strain F1 and *A. niger* VKMF-1119

**Fig. 8.**
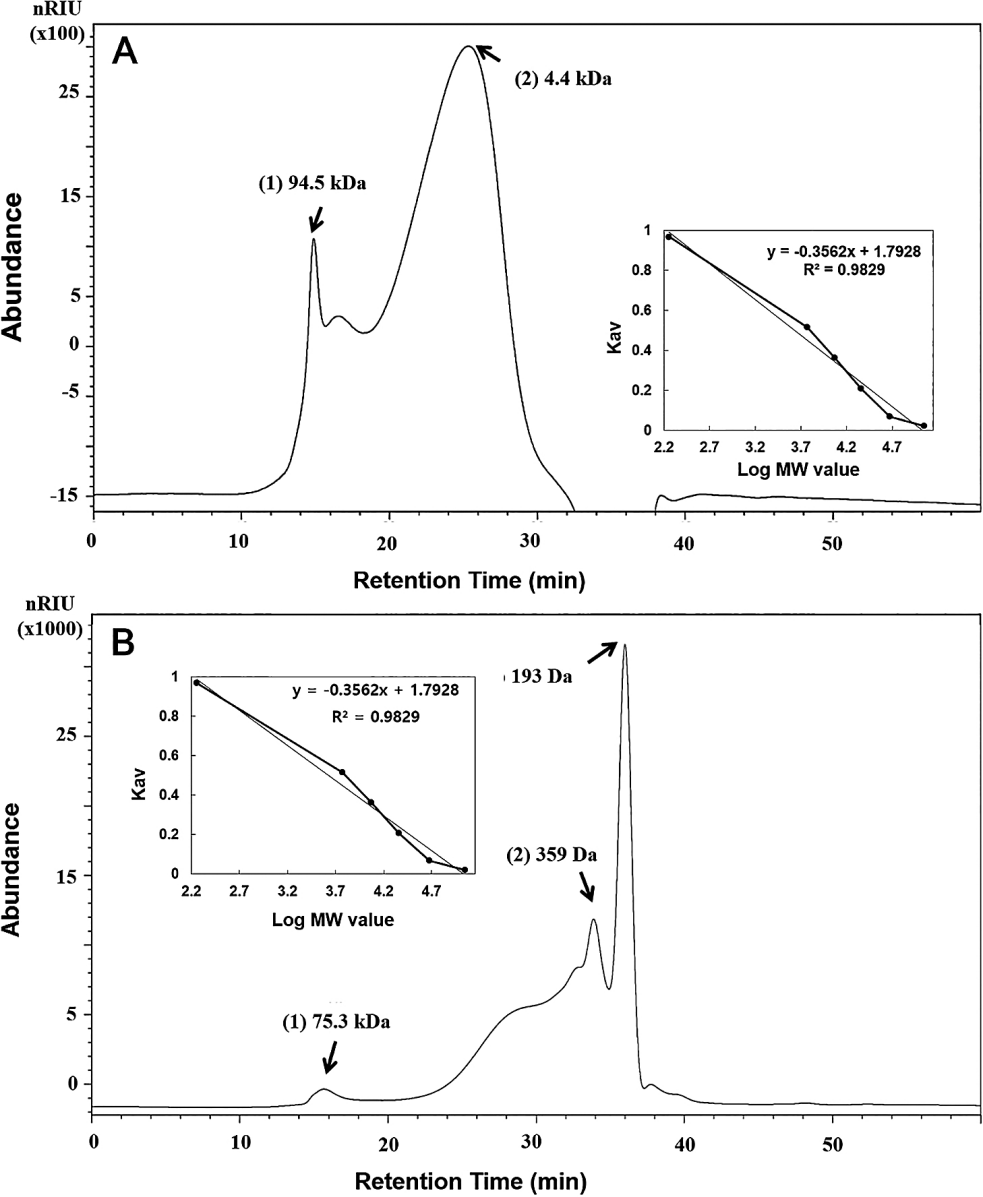
Molecular weights of EPSs from *Cladosporium* sp. strain F1 (A) and *Aspergillus niger* VKMF-1119 (B). The calibration curve equation is Log MW=1.7928–0.3562x, R^2^=0.9829, where MW is the molecular weight of the standard dextran and x is the retention time (inset).

The MW, sugar compositions, and structures of EPSs were analyzed using HPSEC, HPLC, and methylation ana­lyses. The MW of the two EPSs markedly differed (Fig. 8). The MW of EPSs from *Cladosporium* sp. strain F1 were 94,900 and 4,400 Da, while those from *A. niger* VKMF-1119 were 75,300, 359, and 193 Da. Overall, *Cladosporium* sp. strain F1 EPSs were richer in high MW polysaccharides than *A. niger* VKMF-1119 EPSs.

Table 1 shows that the relative amounts of neutral sugar and glucose mole (%) in EPSs from *Cladosporium* sp. strain F1 were lower than those from *A. niger* VKMF-1119. However, mannose and galactose mole (%) were high in *Cladosporium* sp. strain F1. The fungal cell wall structure is complex and flexible, being composed of approximately 80% polysaccharides and small amounts of proteins, lipids, pigments, and inorganic salts (Gow *et al.*, 2017). Therefore, these results also suggest that the two EPSs are polysaccharides with glucan and mannan structures released from fungal cell walls under the culture conditions used.

Table 2 shows the glycosidic linkage conformation of polysaccharides in fungal strains, which was detected by the well-established methylation method for elucidating the structures of polysaccharides. EPSs from *Cladosporium* sp. strain F1 contained more than sixteen different types of glycosyl linkages, including 4-linked (44.4%), terminal (13.2%), 3,4-branched (10.2%), 4,6-branched (8.4%), and 3,4,6-branched (5.6%) glucopyranosides. However, EPSs from *A. niger* VKMF-1119 contained more than eleven different types of glycosyl linkages, including 4-linked (38.4%), terminal (29.0%), 4,6-branched (10.2%), 6-linked (6.3%), and 3,4-branched (5.5%) glucopyranosides. Based on these results, both samples appeared to be highly branched glucans; however, the structures of EPSs from *Cladosporium* sp. strain F1 were larger and more branched than those of *A. niger* VKMF-1119. Further comparative research is needed to identify which component or structure in the fungal EPSs of *Cladosporium* sp. strain F1 contributes to the better adsorption of uranium phosphate minerals on fungal hyphae.

While previous studies have generally focused on the biosorption of cationic heavy metal species, such as lead, copper, and cadmium ions, onto a number of negatively charged functional groups in the fungal and/or bacterial cell surface, the biosorption of negatively charged heavy metal species, including oxyanions of chromium and arsenic, has not been examined as extensively (Michalak *et al.*, 2013). Uranyl hydrogen phosphate hydrates, which were a close match mineralogically with the pre-synthesized uranium phosphate minerals used in the present study, were shown to be composed of negatively charged layers (Morosin, 1978; Pozas-Tormo *et al.*, 1986; Tu *et al.*, 2019). Differences in the adsorption capacity of the two fungi to the negatively charged layers of pre-synthesized uranyl hydrogen phosphate hydrates may be explained by the total interaction energy, which is calculated by the repulsive electrostatic force and attractive van der Waals force (Pajerski *et al.*, 2019). These physicochemical parameters are attributed to the different physical and chemical structures of the cell wall and different amounts of extruded EPSs, which also offer many different functional groups, such as carboxyl, hydroxyl, sulphate, phosphate, and amino groups in varying degrees (Yin *et al.*, 2011; Chen *et al.*, 2013; Li *et al.*, 2020). Therefore, as shown in Table 1 and 2, the purified EPSs of *Cladosporium* sp. strain F1 and *A. niger* VKMF-1119 had different compositions and structures, which may partially contribute to the differences observed in the total interaction energy, thereby leading to differences in the adsorption of uranyl hydrogen phosphate hydrates. However, we were unable to identify which functional groups or molecules of cell wall components and EPSs were involved in the adsorption of negatively charged minerals. Further comparative genomic and biochemical research is needed to identify the key biological components that contribute to the preferential adsorption of pre-synthesized uranyl hydrogen phosphate hydrates on *Cladosporium* sp. strain F1.

## Conclusions

The ability of *Cladosporium* sp. strain F1 to remove uranium phosphate minerals was investigated, and the results obtained revealed unusual structures encrusting fungal hyphae with multiple layers. Regardless of mineral shapes and the pH conditions applied, the biosorption capacity of *Cladosporium* sp. strain F1 towards uranium phosphate minerals was superior to that of *A. niger* VKMF-1119. Based on the physical mobility and chemical stability of uranium phosphate minerals under *in situ* oxidizing environmental conditions, *Cladosporium* sp. strain F1 appears to be a better option for collecting and removing uranium from a wide range of pH conditions where large amounts of phosphate are present in the environment.

## Citation

Lee, J., Lee, S. J., Kim, S., Lee, J.-U., Shin, K.-S., and Hur, H.-G. (2021) Layers of Uranium Phosphate Nanorods and Nanoplates Encrusted on Fungus *Cladosporium* sp. Strain F1 Hyphae. *Microbes Environ ***36**: ME21036.

https://doi.org/10.1264/jsme2.ME21036

## Supplementary Material

Supplementary Material

## Figures and Tables

**Table 1.** Compositional analyses of EPSs from *Cladosporium* sp. strain F1 and *Aspergillus niger* VKMF-1119.

**A**	Polysaccharide component	*Cladosporium* sp. strain F1	*Aspergillus niger* VKMF-1119	(%)
	Neutral sugar	93.1±2.1	96.2±4.3	
	Uronic acid	4.8±0.3	3.4±0.2	
	Protein	2.1±0.5	0.4±0.4	
**B**	Neutral sugar			(Mole%)
	Mannose	6.9±0.2	3.6±0.2	
	Glucose	88.1±0.7	96.4±0.2	
	Galactose	5.0±0.5	—	

**Table 2.** Structural analyses of EPSs from *Cladosporium* sp. strain F1 and *Aspergillus niger* VKMF-1119.

Fungal strain	Monosaccharide	Glycosidic linkage	Mole%
*Cladosporium* sp. strain F1	Glucose	Terminal	**13.2**
		2-	1.0
		6-	3.9
		4-	**44.4**
		3,4-	**10.2**
		2,4-	1.9
		4,6-	**8.4**
		3,4,6-	**5.6**
		2,4,6-	0.7
	Mannose	3-	4.0
		2-	1.9
		3,6-	0.7
		2,6-	0.8
	Galactose	3,6-	0.8
		4,6-	1.8
		2,4,6-	0.7
*Aspergillus niger* VKMF-1119	Glucose	Terminal	**29.0**
		2-	1.6
		6-	6.3
		4-	**38.4**
		3,4-	5.5
		2,4-	1.8
		4,6-	**10.2**
		3,4,6-	2.2
	Mannose	3-	2.5
		2-	1.8
		3,6-	0.7
