# Supplementary material for: Layers of Uranium Phosphate Nanorods and Nanoplates Encrusted on Fungus Cladosporium sp. Strain F1 Hyphae
Source: Microbes Environ. 2021 Nov 13;36(4):ME21036. doi: 10.1264/jsme2.ME21036 (PMC8674443; doi:10.1264/jsme2.ME21036)
Supplement: Supplementary file 1 — Supplementary Material [file 36_21036_s1.pdf]

Submission to Microbes and Environments

Supporting Information for

Layers of Uranium Phosphate Nanorods and Nanoplates Encrusted on Fungus *Cladosporium* sp. Strain F1 Hyphae

Jisu Lee[1], Sue Jung Lee[2], Sungho Kim[3], Jong-Un Lee[4], Kwang-Soon Shin[2]*, Hor-Gil Hur[1]*

[1]School of Earth Sciences and Environmental Engineering, Gwangju Institute of Science and Technology, Gwangju 61005, Republic of Korea,

[2]Department of Food Science and Biotechnology, Kyonggi University 154-42, Gwanggyosan-ro, Youngtong-gu, Suwon, Gyeonggi 16227, Republic of Korea,

[3]GIST Central Research Facilities, Gwanju Institute of Science and Technology, Gwangju 61005, Republic of Korea, and

[4]Department of Energy and Resources Engineering, Chonnam National University, Gwangju 61186, Republic of Korea

* To whom correspondence should be addressed: Hor-Gil Hur (Tel: +82-062-715-2437; Fax: +82-062-715-2437; E-mail: hghur@gist.ac.kr) and Kwang-Soon Shin (Tel: +82-031-249-9655; Fax: +82-031-249-9650; E-mail: ksshin@kyonggi.ac.kr).

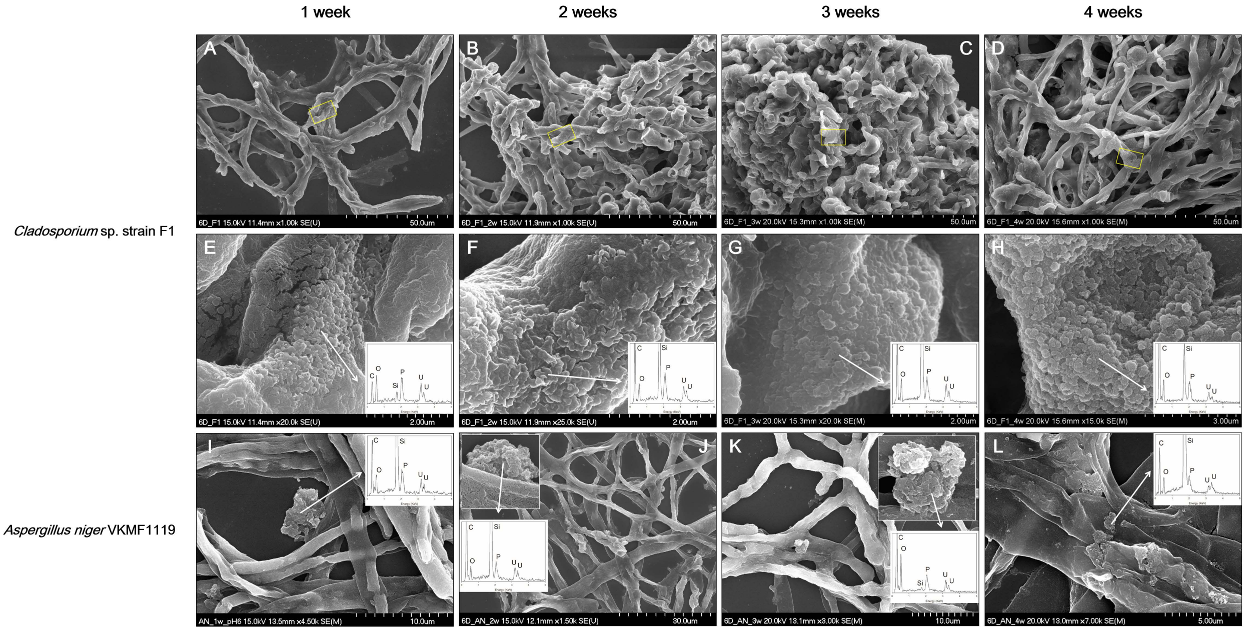

Figure S1. SEM images with EDX results (inset) of the uranium phosphate precipitates adsorbed on the hyphae of *Cladosporium* sp. strain F1 (A - H) and *A. niger* VKMF-1119 (I - L) after biomineralization at pH 6. The fungal biomass was collected at 1 (A, E, and I), 2 (B, F, and J), 3 (C, G, and K), and 4 weeks (D, H, and L).

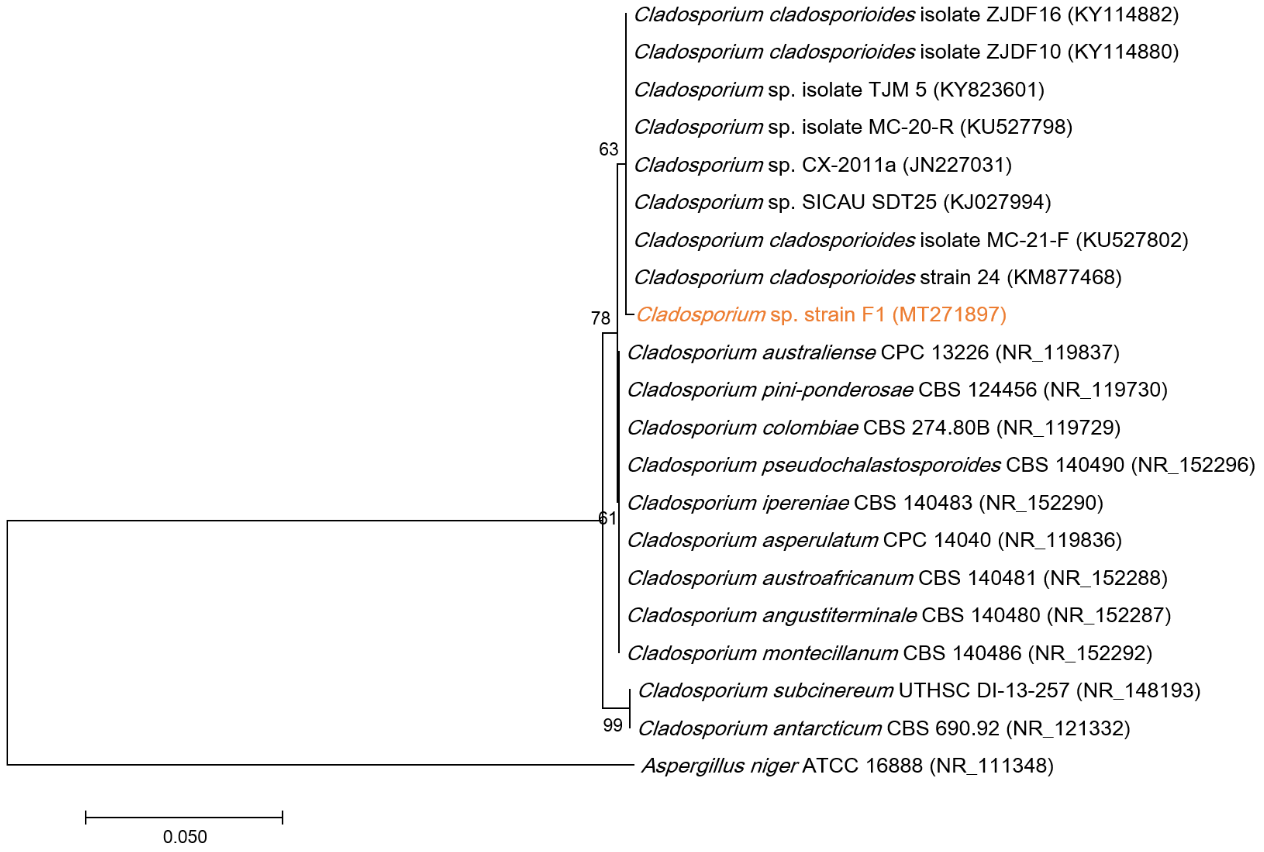

Figure S2. Phylogenetic tree of the isolated fungus by ITS sequence analyses. The strain was identified as genus *Cladosporium*. The tree was inferred by using Maximum likelihood treeing algorithm and bootstrap values were calculated from 1,000 replicates. *Aspergillus niger* ATCC 16888 was used as the outgroup. Bar, 0.05 substitution per nucleotide position.
